# Why non-invasive maternal hemodynamics assessment is clinically relevant in early pregnancy: a literature review

**DOI:** 10.1186/s12884-016-1091-9

**Published:** 2016-10-12

**Authors:** Sharona Vonck, Anneleen Simone Staelens, Ine Bollen, Lien Broekx, Wilfried Gyselaers

**Affiliations:** 1 Faculty of Medicine and Life Sciences, Hasselt University, Hasselt, Belgium; 2 Department of Obstetrics & Gynecology, Ziekenhuis Oost-Limburg, Schiepse Bos 6, 3600 Genk, Belgium; 3 Department Physiology, Hasselt University, Hasselt, Belgium

**Keywords:** Maternal hemodynamics, First trimester, Hypertension, Reference values, Early pregnancy

## Abstract

**Background:**

The maternal cardiovascular system adapts quickly when embryo implantation is recognized by the body. Those adaptations play an important role, as a normal cardiovascular adaptation is a requirement for a normal course of pregnancy. Disturbed adaptations predispose to potential hypertensive disorders further in pregnancy [1–3]. This report aims to briefly inform the obstetricians, general practitioners and midwives, who are the key players in detecting and treating hypertensive disorders during pregnancy.

**Methods:**

The PubMed database was used as main tool to find studies involving clearly defined first trimester hemodynamic changes in normal pregnancies and hypertensive pregnancies. In addition, the bibliographies of these studies were investigated for further relevant literature.

**Results:**

A comprehensive overview is given concerning the normal adaptations in the cardiovascular tree in a first trimester pregnancy. Additionally, signs of abnormal cardiovascular changes observed in first trimester are described together with the normal reference range for each non-invasive, easily applicable technique for maternal hemodynamics assessment.

**Conclusions:**

With a combination of techniques, it is possible to integrate and evaluate the maternal heart, veins and arteries at 12 weeks of pregnancy. Applying those techniques into the daily clinic opens perspectives to prevention and prophylactic treatment, aiming for a reduction of the risk for hypertension during pregnancy.

## Background

A normal pregnancy is characterized by multiple cardiovascular adaptations to provide optimal conditions for fetal growth and development. Most of these changes are induced shortly after embryo implantation and thus well before the exponential increase of the fetal and placental demands for oxygen and nutrients, predominantly occurring in the second half of pregnancy. Most circulatory functions have changed significantly by the end of the first trimester [1].

A maladaptation in any step of the process changes the structural, physiological or metabolic environment of the fetus. The genetic program of the fetus induces phenotypic adaptions to the current environment, which influences the fetus’ cardiovascular system in later life [2]. Additionally, those disturbed physiological processes predispose to pregnancy complications, such as preeclampsia and fetal growth restriction [3].

This review provides a summary of the most relevant cardiovascular changes in the first trimester of a normal pregnancy, as reported in diverse studies done in early pregnancy. Adaptations are presented and discussed at each part of the cardiovascular tree, the arteries, veins and heart. Additionally, a comprehensive overview is given about the pathology-specific features of hypertensive disorders, measurable in the first trimester, together with the normal values of each non-invasive, easily applicable technique.

## Pre-pregnancy cardiovascular function

The cardiovascular function in women varies with the menstrual cycle. In the luteal phase, heart rate, plasma volume, cardiac output (CO), central and peripheral arterial compliance and distensibility have increased relative to the follicular phase. Meanwhile, mean arterial pressure (MAP) has decreased together with the vascular resistance. Chapman suggests that the fall in peripheral vascular resistance is the primary event in the luteal phase. Afterwards, the MAP decrease initiates a hemodynamic compensation through a rise in CO. Due to a dilated vascular system, the renal plasma flow and glomerular filtration rate increase and an activation of the renin-angiotensin-aldosterone system (RAAS) during the luteal phase is seen [4, 5].

This cyclic pattern resembles roughly to the simultaneous hormonal variations occurring during the menstrual cycle, which suggests prudently a role for estrogen in modulating the vascular smooth muscle tone. Estrogen has been shown to enhance endothelial function, dilate arterioles, vascular smooth muscle cell proliferation, inhibit fibroblast and collagen accumulation in the aortic wall [6]. The exact mechanism by which estrogens modulates arterial function has been explored in animal studies: vascular smooth muscle cells have estrogen receptors [7] and estrogen administration induces vasodilatation [8]. It is however difficult to postulate a direct relation between estrogen and cardiovascular events, as other factors (like nitric oxide, relaxin, vascular endothelial growth factor, prostanoid metabolites, adhesion molecules, etc.) also vary during the menstrual cycle. It is probably a complex cascade of events which initiates the primary vasodilation [9].

Early pregnancy is characterized by vascular relaxation along with increased arterial compliance and distensibility. The pattern of cardiovascular changes in the luteal phase relative to the follicular phase of the menstrual cycle resemble those in early pregnancy. From conception until the 7^th^ week of pregnancy, the maternal endocrine environment is dominated by the extended function of the corpus luteum, which is activated through the release of large amounts of human chorionic gonadotropin (hCG). Consequently, the hemodynamic and hormonal changes seen in the first weeks of gestation are independent of a functioning fetal-maternal-placental unit [5].

## Cardiovascular changes

### 5 weeks of gestation

Doppler echography at the level of the pulmonary, mitral and aortic valves at 5 weeks pregnancy showed consistently higher values for heart rate and cardiac output relative to the prepregnant state (luteal phase) [10, 11], whereas others reported a rise in cardiac output occurring later on in the first trimester [12] or not at all [13]. This discrepancy might be explained by the absence of a pre-pregnancy baseline [12] or the different measuring time points and design of the study [13]. Both heart rate and cardiac output increase steadily in the course of the first trimester [10, 12].

Cardiac output depends on heart rate, contractility, preload and afterload. Consequently, the initial increase in cardiac output is achieved by a rise in a higher heart frequency [10, 14], simultaneously with the reduction in peripheral vascular resistance [10–12]. Peripheral vascular resistance decreases abruptly by 20–25 % at 5 weeks compared to the beginning of the menstrual cycle [11], most likely in concert with an abrupt fall in plasma osmolality [15]. Also stroke volume and plasma volume are reported to be increased at week 5, indicating that all these adaptive changes contribute to the prolonged institution of a high cardiac output and low peripheral resistance circulation [10, 16].

### 6 weeks of gestation

At 6 weeks pregnancy, the magnitude of systemic vasodilatation had increased relative to 5 weeks [17] with lower blood pressures (systolic + diastolic, brachial and central) than before conception, occurring secondary to the peripheral vasodilation. This event arises together with an additional fall in pulse wave velocity, a parameter correlating with aortic stiffness [1, 12, 18]. Systolic and diastolic blood pressure continue to decrease until 24 weeks of gestation, because the CO incline is not sufficient to prevent the blood pressure fall as blood pressure is the product of cardiac output and total peripheral vascular resistance [17].

Shortly after implantation, the corpus luteum releases plasma relaxin, which is 3–4 fold higher at 6 weeks than during the menstrual cycle. Relaxin circulates in the maternal blood and rapidly reaches a plateau at the end of the first trimester. It is suggested to play a role in the vasodilation pathway, but the exact pathway is still unclear. The vasodilatory response of relaxin is mediated by its major receptor (relaxin/insulin-like family peptide 1 receptor, RFXP1) and depends on duration of hormone exposure. The rapid vasodilatory responses are performed through the activation of the pathway stimulating nitric oxide synthase. The sustained vasodilatory response relies on vascular endothelial and placental growth factors and the arterial gelatinase activity [19].

Renal hemodynamics are also characterized by a marked fall in renal vascular resistance, giving rise to a correspondingly increase in renal perfusion [20]. The latter induces a ≈ 50 % rise in glomerular filtration rate (GFR) accompanied by a small decline in filtration fraction. Meanwhile, the low blood pressures activate renin-angiotensin-aldosterone system (RAAS), which increases plasma renin activity and plasma aldosterone. Angiotensin II induces constriction of the renal blood vessels, excretion of the vasopressin hormone and aldosterone/adrenaline/noradrenaline production. This mechanism, together with the higher aldosterone production, leads to sodium and water reabsorption. The vasopressin hormone prevents re-excretion of the water [21]. This causes the expansion of plasma volume, hemodilution, a higher renal plasma flow and a higher cardiac output [18, 22]. Cardiac preload (i.e. venous return) increases in concert with the plasma volume expansion. This improves cardiac filling during diastole, which raises the efficiency of the Frank-Starling mechanism. Due to concomitant fall in cardiac afterload, a higher stroke volume arises and the elevated cardiac output is preserved, which anticipates an adequate fetal and placental supply of oxygen and nutrients throughout the course of pregnancy. These changes occur long before the uteroplacental circulation has become an important part of the systemic circulation [18, 23, 24].

### 7 weeks of gestation

At 7 weeks of gestation an increasing distensibility and compliance of the arterial and venous compartment has been observed [11, 25, 26]. It is suggested that the arterial compliance increases due to relaxation of the muscular wall of the vessels [11, 26]. The decrease in peripheral vascular resistance causes a decrease in diastolic pressure, but the presence of a higher arterial compliance counterbalances this event, assuring normalized perfusion pressure to coronaries and vital organs [26, 27]. Both events may play a role in enhancing the left ventricular performance [25]. A higher venous compliance increases the capacity of the venous compartment to accommodate a larger reserve volume in the splanchnic system, which is also necessary to preserve the cardiac output during pregnancy [28]. The venous compliance keeps on rising by advancing pregnancy [29].

### 8 weeks of gestation

Due to the increasing cardiac preload together with the lower afterload early in pregnancy, stroke volume and cardiac output keep on rising during the first trimester [10]. The continuous increase of cardiac output is from this moment not only caused by a higher heart rate, but is supported by the increase in stroke volume together with the observed anatomic increase of the heart surface area [12, 14]. The left atrial diameter increases together with the left ventricular diastolic dimension, which is a measurement of the preload [12]. The systolic dimension of the left ventricle and the ventricle wall thickness were also significantly increased [10, 14]. These changes enhance the performance of the total left ventricle [12]. The aortic, pulmonary and mitral velocities increase steadily and become significantly higher in week 8 as compared to pre-pregnancy values [10]. The onset of intervillous perfusion with maternal blood, starting to develop at ≈ 8 weeks, is not only associated with a rise in the intervillous oxygen tension, but also with a rise in the circulating levels of a number of placental biochemical markers for oxidative stress [30].

### 9 to 12 weeks of gestation

At 8 weeks, the first morphological changes have been reported in multiple studies. At 12 weeks, Doppler echocardiography indicated more advanced changes in the heart surface area: the mitral, aortic and pulmonary valve areas increase, together with diastolic dimensions, left atrial dimensions, total left ventricle wall thickness and left ventricle mass [10]. Left ventricular diastolic and systolic volumes are both increased comparing to pre-pregnancy, but not yet significant according to the first trimester study of Del Bene [13]. The increase of the cardiac dimensions are probably a consequence of the persistent increase of venous return and cardiac filling pressure, which can be expected to improve the cardiac performance continuously [10]. Cardiac output, blood volume and plasma volume are highest at 12 weeks in the first trimester, which might explain why most morphological heart changes only become significant around 12 weeks [18]. The progressive enlargement of the heart surface area represents the typical gestational phenomenon ‘myocardial eccentric hypertrophy’. This is a reversible hypertrophy without any long term cardiac consequences [10, 31]. Interestingly, all these pregnancy-related cardiac changes are identical to patients with a chronic volume overload state and in isotonic-exercising athletes (swimming, running) 10 to 12 weeks after the start of the training program: higher left ventricle volume, left ventricle mass and ventricular performance [32].

In the first trimester, renal plasma flow (RPF) reaches a peak at 12 weeks. Afterwards RPF decreases. Glomerular filtration rate had increased steadily in the first trimester, continues to increase in the second trimester but tends to decline again in the third trimester. Atrial natriuretic peptide (α-ANP) is significantly raised at week 12, and keeps increasing even more afterwards. It has diuretic, natriuretic and vasodilator properties and might play an important role in volume homeostasis [18]. α-ANP is considered not active in early pregnancy because plasma concentrations remain low during the first weeks, but increase by 35 % in the second trimester in association with the plasma volume increase. This suggests that plasma α-ANP changes are secondary to the plasma volume increase, rather than playing a primary role in the hemodynamic changes [12, 18].

In the second half of the first trimester, retrograde trophoblast invasion into the spiral artery together with a lower responsiveness to vasoconstrictor agents may explain the further rise in uteroplacental vascular compliance, as the resistance index (RI) and pulsatility index (PI) of the uterine artery decrease when gestational age increases. RI and PI are representative for the resistance in the uterine arteries. There exists an inverse relation between RI or PI with ongoing trophoblastic invasion into the spiral arteries [33, 34] Intervillous blood flow becomes only detectable from 12 weeks onwards, because trophoblastic migration is only observed from week 10, and a consecutive formation into the myometrium of the spiral arteries starts from week 14. This implies that physiological changes are completed and heart/vessels are adapted morphologically. Spiral arteries remodeling induces the change from high-resistance flow to low-resistance flow [30]. Also further studies have shown a continuous decrease of resistance and pulsatility indices between 12 and 16 weeks [35–37] (Table 1).

**Table 1:** Overview of all early cardiovascular changes in normal pregnancy

		Early first trimester			Late first trimester
		5 weeks	6 weeks	7 weeks	8–12 weeks
Arteries	Total Peripheral Resistance (TPR)	↓			
	Pulse Wave Velocity (PWV)		↓		
	Aorta Compliance			↑	
	Blood Pressure		↓		
	Blood Velocity				↑
Heart	Heart Rate (HR)	↑			
	Stroke Volume (SV)	↑			
	Cardiac Output (CO)	↑			
	Cardiac Filling Pressure		↑		
	Inotropy				↑
	Heart Surface Area				↑
	Atrial-Ventricular Dimensions				↑
	Ventricular Wall				↑
Veins	Venous Return		↑		
	Distensibility			↑	
	Capacitance			↑	
Hematology	Plasma Volume	↑			
	Plasma Osmolality	↓			
	Relaxin		↑		
Kidney	Glomerular Filtration Rate (GFR)		↑		
	Renal Plasma Flow (RPF)		↑		
	Renal Vascular Dilatation		↓		
	Filtration Fraction		↓		
	Renin Angiotensin Aldosterone System (RAAS)		↑		

## Pathological signs in the first trimester

The cardiovascular system in patients with hypertensive disorders differs from the normal maternal cardiovascular system. Focusing on hemodynamic characteristics could help to understand the physiological maladaptations in the first trimester.

### Arteries

#### Peripheral arteries

Higher uterine pulsatility indices in early preeclampsia than uncomplicated pregnancies have been reported in the early pregnancy (2.35 vs. 1.79), which is a measurement for impaired placentation and arterial stiffness [38]. Additionally, placental volume was shown to be smaller too in (early) preeclamptic patients (60 vs. 43 cm^3^) [38]. In patients developing late preeclampsia however no aberrant uterine pulsatility indices or placental volume were found [38]. There is a direct relation between resistance, arterial stiffness and endothelial dysfunction, which clinically presents in high blood pressures. This is in congruence with measurements for total vascular resistance, which was shown to be higher in the late preeclamptic group (1105 dyne*sec*cm^−5^ versus 1260 dyne*sec*cm^−5^), measured as [MAP (mmHg)/CO (L/min) x 80] [39]. Another measurement for arterial stiffness is the pulse wave velocity (PWV) and augmentation index (AIx). Already at the end of the first trimester, the pulse wave velocity and augmentation index in the brachial artery is significantly higher in the hypertensive group (7.47 m/s vs. 6.55 m/s (PWV); 13.2 % vs. 10.6 % (AIx)) [40]. Peripheral higher blood pressures (diastolic, systolic, mean arterial pressure) could be measured (123 vs. 114 mmHg (SBP); 82 vs. 75 mmHg (DBP); 92 vs. 85 mmHg (MAP)) in an eventual late hypertensive group [41]. All those results indicate a stiffer peripheral arterial system already in the first trimester of pregnancies destined to develop pre-eclampsia.

#### Central arteries

Additionally, the early gestational central systolic blood pressure is also higher in the preeclamptic group (122 mmHg vs. 108 mmHg) [40]. First trimester aorta flow parameters (velocity index (VI), acceleration index (ACI) and heather index (HI)), which are directly related to systolic function and aorta compliance, are shown to be different between normal and pathological pregnancies (VI: 72 vs. 57 1/1000/s; ACI: 133 vs. 108 1/100/s^2^; HI: 23.1 vs. 17.3 Ω/s^2^). Lower values, as seen in the late hypertensive group, reflect a poor ventricular ejection and thus systolic dysfunction already present in the first trimester [41].

### Heart

Khaw et al. investigated different cardiovascular functions at 11–14 weeks between uncomplicated pregnancies, eventual preeclamptic pregnancies with appropriate for gestational age babies (AGA) and preeclamptic patients with small for gestational age babies (SGA). Cardiac output, cardiac index and stroke volume were significantly higher in the preeclamptic group with AGA than the uncomplicated group (6.2 l/min, 3.3 l/min/m^2^, 87.9 ml versus 4.9 l/min, 2.9 l/min/m^2^, 67 ml resp.) [39, 42]. A higher mitral valve annulus shortening is also found in this pathological group (15.5 mm versus 17.8 mm), supporting this suggestion. Preeclampsia with SGA babies have no aberrant cardiac output, cardiac index or stroke volume parameters (4.9 l/min, 2.8 l/min/m^2^, 66.3 ml resp) compared to the uncomplicated group [39]. Further, higher values are observed for mitral valve E-wave velocity (82.4 mm/s versus 95.7 mm/s) in the late preeclamptic group. Mitral valve E-wave velocity measures the diastolic function, which is the passive filling of the ventricle. Khaw et al. also measured the mitral valve A-wave velocity (representation of the active filling during atrial systole), which did not differ significantly between the 2 groups in this study [39]. Both velocities are strongly dependent from physiological changes in heart rate, preload, left ventricular compliance and contractility. An initial rise in the E/A ratio is consistent with an initially impaired adaptation of cardiac diastolic function, which is already present in a first trimester measurement in the hypertensive group. An increase of more than 7 % at 12 weeks indicates an important higher risk for pre-eclampsia [43].

### Veins

Also venous impedance is observed to be higher in pathological pregnancies than normal pregnancies. A high venous impedance is accompanied with a low venous distensibility, a higher venous pressure, a lower venous capacity and a smaller increase in plasma volume [44]. However, venous hemodynamic function is shown to be normal in the first trimester, but becomes abnormal during the second trimester in the pregnancies destined to develop preeclampsia [41].

### Hematology

Plasma volume increase is very important for normal fetal development. Maladaptation of the cardiovascular system can obstruct a necessary increase in plasma volume. There is a correlation between maternal cardiac output and the growth of the fetus, already present in the first trimester [45]. The link between cardiac output and birth weight percentiles is associated with the grade of heart, vein and artery dysfunction because those are the driving forces of the cardiac output [45].

### Kidney

A direct consequence of a high vascular resistance and high blood pressure is the decrease in renal perfusion and glomerular filtrate rate. The renal clearance will decrease, which leads to a higher plasma concentration of uric acid. Preeclampsia is characterized by proteinuria, which possibly relates to a higher capillary pressure in the glomerular filter. This induces a lower tubular absorption, contributing to glomerular endotheliosis. However, no studies are found concerning this topic in first trimester [46].

Aberrant hemodynamic characteristics may already be evident in the first trimester, however not always measurable. More and more researchers focus on well-defined subject groups, as the pathophysiology between gestational hypertension, early preeclampsia and late preeclampsia with and without intra uterine growth restriction have important hemodynamic differences.

## Techniques: normal values

To assess abovementioned maternal cardiovascular adaptations, safe, easily accessible and non-invasive first trimester techniques are useful, which have the potential to evaluate the different compartments of the maternal cardiovascular system.

A Doppler ultrasound of the uterine artery is a widely used, non-invasive technique to measure the pulsatility index (PI; [peak systolic flow – end diastolic flow/mean flow]) and the resistance index (RI; [peak systolic flow – end diastolic flow/peak systolic flow) from the flow velocity waveforms of the uterine arteries. Normal values vary between 0.56 and 0.76 for RI, and minimum 1.11 and maximum 2.08 for PI [33, 35, 39–41, 47, 48]. Low end-diastolic velocities and an early diastolic notch are typical waveforms in the first trimester pregnancy, indicating a high uterine resistance [47, 49, 50]. Other parameters reflecting arterial stiffness is the pulse wave velocity, measured from artery pulse waveforms in the brachial [40, 51, 52] or radial artery [53]. SphygmoCor (West Ryde, Australia), Complior (Vincennes, France) or Arteriograph (TensioMed, Budapest, Hungary) are the three techniques used for pulse wave analysis. The normal pulse wave velocity ranges between 6.13 and 8.13 m/s. PI, measured with the artery pulse waveform technique, lies between 1.4 and 2.08 [40, 51]. In preeclampsia, PI, RI and pulse wave velocity are considerably higher [38–41, 53–55].

The combination of electrocardiogram and Doppler ultrasound is used to investigate the maternal venous system (renal interlobular veins and hepatic veins), using venous impedance indices and the ‘venous pulse transit time’ (VPTT). Tomsin et al. reported a gradual rise of the VPTT in normal pregnancy and a significantly shorter VPTT in preeclamptic patients, which illustrates a venous hemodynamic dysfunction in preeclampsia [56, 57]. Also aberrant venous impedance indices (Hepatic Vein Index (HVI), Renal Interlobar Vein Index (RIVI)) are seen in the active phase of preeclampsia. Compared to normal pregnancies, in the preeclamptic group at 12 weeks, the venous function seems to be normal [VPTT kidney: 0.24–0.35 ms; VPTT liver: 0.13–0.23 ms; HVI: 0.7–1.6; RIVI: 0.38–0.56] [41].

Aortic Doppler flow measurements estimate the aortic valve area, cardiac output and stroke volumes. The aortic area has a normal range from 3.85 to 3.94 cm^2^. Stroke volume with this technique is reported between 81.5 and 84.1 ml, and cardiac output between 6.61 and 6.83 l/min [10]. In (late) preeclampsia, stroke volume and cardiac output are higher [39, 42]. Aortic valve area uses calculations from the aortic diameter, which is reported to be higher in preeclampsia in the first trimester [58].

Echocardiography is the preferred technique to evaluate the systolic and diastolic function of the heart during the complete course of pregnancy. Cardiac output, stroke volume (systolic function); E-wave and A-wave (diastolic function) and mitral valve annulus shortening measurement are parameters measured with this technique. Diastolic function has an E-wave range from 68.8 to 96 mm/s, and an A-wave range from 40.5 to 57.3 mm/s. The mitral valve annulus is found to be normal between 13.5 and 17.5 mm [39]. Robson et al. measured also left ventricle End-Systolic Dimension (ESD), ranging from 2.85 to 2.91 cm, and End-Diastolic Dimension (EDD), ranging from 4.63 to 4.69 cm. The Left Atrial Dimension (LAD) ranges between 3.26 and 3.36 cm. Further, the thickness of the left ventricle wall is measured, as 1.56–1.62 cm, and the mass: 134–140 g. The ejection fraction is 75.7–76.9 %, all parameters measured around 12 weeks [10]. In preeclampsia, increased mitral valve shortening and E-wave are reported, but significant differences in ESD, EDD, LAD, left ventricular wall thickness and mass were not observed [43].

Other systolic parameters can be measured by impedance cardiography, which monitors the cardiovascular changes by transmitting an electrical current with high frequency and low amplitude through the maternal thorax. Another non-invasive method is the USCOM (Sydney, Australia) device which measures the velocity of the aortic/pulmonary blood flow, used to calculate a time-velocity integral, and as such basically is a derived echocardiography technique. In these cases, cardiac output and stroke volume seem to have an overestimation with those two techniques relative to echocardiography measurements as the normal range for CO is reported around 6.48–7.89 l/min, and for SV 72.62–94.62 ml. Echo cardio measurements are lower: CO 4.3–5.5 l/min, and SV 55.3–78.7 ml. Non-Invasive Cardiac Output Monitor (NICOM, Cheetah Medical, USA) also measures HR, CO and SV. It is based on a thoracic bioreactance technology, but no published studies on patients in the first trimester of pregnancy were traced. It is still quite controversial using non-invasive techniques in clinics, as the reliability compared to the golden standard echocardiography is highly discussed in literature. However, using only one technique with its method-specific reference values eliminates possible over- or underestimation [39, 41, 55, 59]. With the use of the formula: [(MAP(mmHg)/CO(L/min) x 80], total peripheral resistance (dyne*sec*cm^−5^) can also be calculated (1015–1212) [10, 39]. With the USCOM device, this parameter is automatically measured using a non-reported formula and the range is found to be slightly lower (940.87–1118.53) [55]. Impedance cardiography additionally provides some important aorta flow parameters: aorta velocity index (1/1000/s), acceleration index (1/100/s^2^) and heather index (Ω/s^2^). In the first trimester, those parameters have an important implication. Normal values are seen between 62 and 82; 107 and 156; 19.2 and 26.9 respectively. In preeclampsia, aortic flow measurements are in a lower range [41].

Studies using echocardiography, impedance cardiography and USCOM additionally measured the blood pressures. Normal systolic blood pressures at 12 weeks vary from 107 to 122 mmHg, diastolic blood pressures range from 65 to 77 mmHg. The mean arterial pressure lies between 77 and 86 mmHg. Heart rates range in the first trimester between 76 and 92 [10, 39, 41, 55]. In preeclampsia, first trimester blood pressures and heart rate are already slightly higher than normal [39, 41, 53–55] (Table 2).

**Table 2:** Normal reference values of different non-invasive techniques

	Pulse Wave Doppler [10, 33, 35, 39–41, 47, 48]	Artery Pulse Waveforms [40, 51, 52]	Echocardiography [10, 39]	Impedance Cardiography [41]	USCOM [55]
Peripheral arteries	PI: 1.11–2.08 RI: 0.56–0.76	PI: 1.4–2.08 PWV (m/s): 6.13–8.13	MAP (mmHg): 73–83 SBP (mmHg): 102–113 DBP (mmHg): 58–67 TVR (dyne*sec*cm^−5^): 1015–1212	MAP (mmHg): 81–90 SBP (mmHg): 106–123 DBP (mmHg): 71–81	SBP (mmHg): 114–130 DBP (mmHg): 67–82 TVR (dyne*sec*cm^−5^): 941–1118
Central arteries	Aortic area (cm^2^): 3.85–3.94	SBP (mmHg): 101–117		ACI (1/100/s^2^): 107–156 VI (1/1000/s): 62–82 HI (Ω/s^2^): 19.2–26.9	
Heart	CO (l/min): 6.61–6.83 SV (ml): 81.5–84.1		CO (l/min): 4.3–5.5 SV (ml): 55.3–78.7 HR (bpm): 73–85 MVAS (mm): 13.5–17.5 E-wave (mm/s): 68.8–96 A-wave (mm/s): 40.5–57.3 ESD (cm): 2.85–2.91 EDD (cm): 4.63–4.69 LAD (cm): 3.26–3.36 LV wall thickness (cm): 1.56–1.62 LV mass (g): 134–140 EF (%): 75.7–76.9	CO (l/min): 6.2–8.2 SV (ml): 66–86 HR (bpm): 87–104	CO (l/min): 6.8–7.6 SV (ml): 79.23–103.23 HR (bpm): 69–89
Veins	RIVI: 0.38–0.56 HVI: 0.7–1.6 VPTT (kidneys) (ms): 0.24–0.35 VPTT (liver) (ms): 0.13–0.23				

*MAP* Mean Arterial Pressure, *SBP* Systolic Blood Pressure, *DBP* Diastolic Blood Pressure, *TVR* Total Vascular Resistance, *CO* Cardiac Output, *SV* Stroke Volume, *HR* Heart Rate, *MVAS* Mitral Valve Annulus Shortening, *E-wave* E-wave velocity, *A-wave* A-wave velocity, *ESD* End-Systolic Dimension, *EDD* End-Diastolic Dimension, *LAD* Left Atrial Dimension, *LV* Left Ventricle, *EF* Ejection Fraction, *ACI* Acceleration Index, *VI* Velocity Index, *HI* Heather Index, *PI* Pulsatility Index, *RI* Resistivity Index, *RIVI* Renal Interlobar Vein Index, *HVI* Hepatic Vein Index, *VPTT* Venous Pulse Transit Time, *PWV* Pulse Wave Velocity

## Application of techniques

Together with gaining basic knowledge about the normal physiological cardiovascular changes in the first trimester of pregnancy, all techniques discussed above allow investigating a population of 12 week pregnant women non-invasively, which opens a window to screen for hypertensive disorders. With a combination of techniques, it is possible to integrate and evaluate the maternal heart, veins and arteries at 12 weeks of pregnancy [41]. In this review, normal values are summarized which opens easily future perspectives to search for aberrant cardiovascular signs in each part of the cardiovascular tree of the doctors patients. Screening for hypertensive disorders is very useful as an early stratification into a high risk vs. a low risk group is possible. Possible follow-up or treatment strategies could be based on prescribing low-dose aspirin before 16 weeks of gestation, which is associated with a 50 % reduction of PE risk and 80 % of early PE [60, 61], paying attention to regular physical activity during pregnancy [62] or remote blood pressure monitoring [63]. Applying those techniques into the daily clinic opens perspectives to prevention and prophylactic treatment, aiming for a reduction of the risk for hypertension during pregnancy. In the long run, it may reduce significantly the maternal and neonatal morbidity and mortality associated with hypertensive disorders [64, 65]. It should however be emphasized that in this review all described hemodynamic changes with corresponding normal or hypertensive values reflect average values, which are subject to individual variability.

## Conclusion

Many recent studies disclose the fact that initiation of placentation is not a necessary step for the normal or pathological hemodynamic alterations in a pregnancy, as placentation starts later than the very first maternal hemodynamic changes. This emphasizes the important role of the maternal cardiovascular system during gestation, with a normal hemodynamic adaptation as a requirement for a normal course of pregnancy. Today, many non-invasive techniques allow a detailed assessment of maternal cardiovascular adaptations which opens perspectives to early postconceptional screening for gestational hypertensive disorders.

## Abbreviations

ACI: Acceleration index
AGA: Appropriate for gestational age
AIx: Augmentation index
ANP: Atrial natriuretic peptide
CO: Cardiac output
DBP: Diastolic blood pressure
EDD: End-diastolic dimension
ESD: End-systolic dimension
GFR: Glomerular filtration rate
hCG: human chorionic gondadotropin
HI: Heather index
HVI: Hepatic vein index
LAD: Left atrial dimension
MAP: Mean arterial pressure
PI: Pulsatility index
PWV: Pulse wave velocity
RAAS: Renin – Angiotensin – Aldosterone – System
RFXP1: Relaxin/Insulin-like family peptide 1 receptor
RI: Resistivity index
RIVI: Renal interlobal vein index
RPF: Renal plasma flow
SBP: Systolic blood pressure
SGA: Small for gestational age
VI: Velocity index
VPTT: Venous pulse transit time

## References

[1] Mahendru AA, Everett TR, Wilkinson IB, Lees CC, McEniery CM (2012). Maternal cardiovascular changes from pre-pregnancy to very early pregnancy. J Hypertens.

[2] Santos MS, Joles JA (2012). Early determinants of cardiovascular disease. Best Pract Res Clin Endocrinol Metab.

[3] Chang J, Streitman D (2012). Physiologic adaptations to pregnancy. Neurol Clin.

[4] Spaanderman ME, Van Beek E, Ekhart TH, Van Eyck J, Cheriex EC, De Leeuw PW (2000). Changes in hemodynamic parameters and volume homeostasis with the menstrual cycle among women with a history of preeclampsia. Am J Obstet Gynecol.

[5] Chapman AB, Zamudio S, Woodmansee W, Merouani A, Osorio F, Johnson A (1997). Systemic and renal hemodynamic changes in the luteal phase of the menstrual cycle mimic early pregnancy. Am J Physiol.

[6] Giannattasio C, Failla M, Grappiolo A, Stella ML, Del Bo A, Colombo M (1999). Fluctuations of radial artery distensibility throughout the menstrual cycle. Arterioscler Thromb Vasc Biol.

[7] Losordo DW, Kearney M, Kim EA, Jekanowski J, Isner JM (1994). Variable expression of the estrogen receptor in normal and atherosclerotic coronary arteries of premenopausal women. Circulation.

[8] Williams JK, Adams MR, Herrington DM, Clarkson TB (1992). Short-term administration of estrogen and vascular responses of atherosclerotic coronary arteries. J Am Coll Cardiol.

[9] Williams MR, Westerman RA, Kingwell BA, Paige J, Blombery PA, Sudhir K (2001). Variations in endothelial function and arterial compliance during the menstrual cycle. J Clin Endocrinol Metab.

[10] Robson SC, Hunter S, Boys RJ, Dunlop W (1989). Serial study of factors influencing changes in cardiac output during human pregnancy. Am J Physiol.

[11] Spaanderman ME, Willekes C, Hoeks AP, Ekhart TH, Peeters LL (2000). The effect of pregnancy on the compliance of large arteries and veins in healthy parous control subjects and women with a history of preeclampsia. Am J Obstet Gynecol.

[12] Duvekot JJ, Cheriex EC, Pieters FA, Menheere PP, Peeters LH (1993). Early pregnancy changes in hemodynamics and volume homeostasis are consecutive adjustments triggered by a primary fall in systemic vascular tone. Am J Obstet Gynecol.

[13] Del Bene R, Barletta G, Mello G, Lazzeri C, Mecacci F, Parretti E (2001). Cardiovascular function in pregnancy: effects of posture. BJOG.

[14] Laird-Meeter K, van de Ley G, Bom TH, Wladimiroff JW, Roelandt J (1979). Cardiocirculatory adjustments during pregnancy -- an echocardiographic study. Clin Cardiol.

[15] Davison JM, Vallotton MB, Lindheimer MD (1981). Plasma osmolality and urinary concentration and dilution during and after pregnancy: evidence that lateral recumbency inhibits maximal urinary concentrating ability. Br J Obstet Gynaecol.

[16] Spaanderman M, Ekhart T, van Eyck J, de Leeuw P, Peeters L (2001). Preeclampsia and maladaptation to pregnancy: a role for atrial natriuretic peptide?. Kidney Int.

[17] Carlin A, Alfirevic Z (2008). Physiological changes of pregnancy and monitoring. Best Pract Res Clin Obstet Gynaecol.

[18] Chapman AB, Abraham WT, Zamudio S, Coffin C, Merouani A, Young D (1998). Temporal relationships between hormonal and hemodynamic changes in early human pregnancy. Kidney Int.

[19] Conrad KP (2011). Emerging role of relaxin in the maternal adaptations to normal pregnancy: implications for preeclampsia. Semin Nephrol.

[20] Weissgerber TL, Wolfe LA (2006). Physiological adaptation in early human pregnancy: adaptation to balance maternal-fetal demands. Appl Physiol Nutr Metab.

[21] Boron WF BE. Integration of Salt and Water Balance. In: Saunders, editor. Medical physiology 2nd ed: Elsevier: 2009. p. 870–2.

[22] Boron WF BE, Jones EE. Both maternal cardiac output and blood volume increase during pregnancy. In: Saunders, editor. Medical Physiology 2nd ed: Elsevier: 2009. p. 1184.

[23] Bernstein IM, Ziegler W, Badger GJ (2001). Plasma volume expansion in early pregnancy. Obstet Gynecol.

[24] Tan EK, Tan EL (2013). Alterations in physiology and anatomy during pregnancy. Best Pract Res Clin Obstet Gynaecol.

[25] Hart MV, Morton MJ, Hosenpud JD, Metcalfe J (1986). Aortic function during normal human pregnancy. Am J Obstet Gynecol.

[26] Poppas A, Shroff SG, Korcarz CE, Hibbard JU, Berger DS, Lindheimer MD (1997). Serial assessment of the cardiovascular system in normal pregnancy. Role of arterial compliance and pulsatile arterial load. Circulation.

[27] Conrad KP, Debrah DO, Novak J, Danielson LA, Shroff SG (2004). Relaxin modifies systemic arterial resistance and compliance in conscious, nonpregnant rats. Endocrinology.

[28] Sakai K, Imaizumi T, Maeda H, Nagata H, Tsukimori K, Takeshita A (1994). Venous distensibility during pregnancy. Comparisons between normal pregnancy and preeclampsia. Hypertension.

[29] Gyselaers W, Mesens T, Tomsin K, Peeters L (2009). Doppler assessment of maternal central venous hemodynamics in uncomplicated-- pregnancy: a comprehensive review. Facts Views Vis Obgyn.

[30] Carbillon L, Challier JC, Alouini S, Uzan M, Uzan S (2001). Uteroplacental circulation development: Doppler assessment and clinical importance. Placenta.

[31] Savu O, Jurcut R, Giusca S, van Mieghem T, Gussi I, Popescu BA (2012). Morphological and functional adaptation of the maternal heart during pregnancy. Circ Cardiovasc Imaging.

[32] Lusiani L, Ronsisvalle G, Bonanome A, Visona A, Castellani V, Macchia C (1986). Echocardiographic evaluation of the dimensions and systolic properties of the left ventricle in freshman athletes during physical training. Eur Heart J.

[33] Alves JA, Silva BY, de Sousa PC, Maia SB, Costa FS (2013). Reference range of uterine artery Doppler parameters between the 11th and 14th pregnancy weeks in a population sample from Northeast Brazil. Rev Bras Ginecol Obstet.

[34] Dugoff L, Lynch AM, Cioffi-Ragan D, Hobbins JC, Schultz LK, Malone FD (2005). First trimester uterine artery Doppler abnormalities predict subsequent intrauterine growth restriction. Am J Obstet Gynecol.

[35] Gomez O, Martinez JM, Figueras F, Del Rio M, Borobio V, Puerto B (2005). Uterine artery Doppler at 11–14 weeks of gestation to screen for hypertensive disorders and associated complications in an unselected population. Ultrasound Obstet Gynecol.

[36] Harrington K, Goldfrad C, Carpenter RG, Campbell S (1997). Transvaginal uterine and umbilical artery Doppler examination of 12–16 weeks and the subsequent development of pre-eclampsia and intrauterine growth retardation. Ultrasound Obstet Gynecol.

[37] Jauniaux E, Jurkovic D, Campbell S (1991). In vivo investigations of the anatomy and the physiology of early human placental circulations. Ultrasound Obstet Gynecol.

[38] Arakaki T, Hasegawa J, Nakamura M, Hamada S, Muramoto M, Takita H (2015). Prediction of early- and late-onset pregnancy-induced hypertension using placental volume on three-dimensional ultrasound and uterine artery Doppler. Ultrasound Obstet Gynecol.

[39] Khaw A, Kametas NA, Turan OM, Bamfo JE, Nicolaides KH (2008). Maternal cardiac function and uterine artery Doppler at 11–14 weeks in the prediction of pre-eclampsia in nulliparous women. BJOG.

[40] Khalil A, Akolekar R, Syngelaki A, Elkhouli M, Nicolaides KH (2012). Maternal hemodynamics at 11–13 weeks’ gestation and risk of pre-eclampsia. Ultrasound Obstet Gynecol.

[41] Oben J, Tomsin K, Mesens T, Staelens A, Molenberghs G, Gyselaers W (2014). Maternal cardiovascular profiling in the first trimester of pregnancies complicated with gestation-induced hypertension or fetal growth retardation: a pilot study. J Matern Fetal Neonatal Med.

[42] De Paco C, Kametas N, Rencoret G, Strobl I, Nicolaides KH (2008). Maternal cardiac output between 11 and 13 weeks of gestation in the prediction of preeclampsia and small for gestational age. Obstet Gynecol.

[43] Sep SJ, Schreurs MP, Bekkers SC, Kruse AJ, Smits LJ, Peeters LL (2011). Early-pregnancy changes in cardiac diastolic function in women with recurrent pre-eclampsia and in previously pre-eclamptic women without recurrent disease. BJOG.

[44] Mesens T, Tomsin K, Staelens AS, Oben J, Molenberghs G, Gyselaers W (2014). Is there a correlation between maternal venous hemodynamic dysfunction and proteinuria of preeclampsia?. Eur J Obstet Gynecol Reprod Biol.

[45] Tomsin K, Mesens T, Molenberghs G, Peeters L, Gyselaers W (2013). Characteristics of heart, arteries, and veins in low and high cardiac output preeclampsia. Eur J Obstet Gynecol Reprod Biol.

[46] Jeyabalan A, Conrad KP (2007). Renal function during normal pregnancy and preeclampsia. Front Biosci.

[47] Plasencia W, Maiz N, Bonino S, Kaihura C, Nicolaides KH (2007). Uterine artery Doppler at 11 + 0 to 13 + 6 weeks in the prediction of pre-eclampsia. Ultrasound Obstet Gynecol.

[48] Ridding G, Schluter PJ, Hyett JA, McLennan AC (2014). Uterine artery pulsatility index assessment at 11–13 weeks’ gestation. Fetal Diagn Ther.

[49] McLeod L (2008). How useful is uterine artery Doppler ultrasonography in predicting pre-eclampsia and intrauterine growth restriction?. CMAJ.

[50] Cnossen JS, Morris RK, ter Riet G, Mol BW, van der Post JA, Coomarasamy A (2008). Use of uterine artery Doppler ultrasonography to predict pre-eclampsia and intrauterine growth restriction: a systematic review and bivariable meta-analysis. CMAJ.

[51] Franz MB, Burgmann M, Neubauer A, Zeisler H, Sanani R, Gottsauner-Wolf M (2013). Augmentation index and pulse wave velocity in normotensive and pre-eclamptic pregnancies. Acta Obstet Gynecol Scand.

[52] Mersich B, Rigo J, Besenyei C, Lenard Z, Studinger P, Kollai M (2005). Opposite changes in carotid versus aortic stiffness during healthy human pregnancy. Clin Sci (Lond).

[53] Khalil AA, Cooper DJ, Harrington KF (2009). Pulse wave analysis: a preliminary study of a novel technique for the prediction of pre-eclampsia. BJOG.

[54] Khalil A, Jauniaux E, Cooper D, Harrington K (2009). Pulse wave analysis in normal pregnancy: a prospective longitudinal study. PLoS One.

[55] Tiralongo GM, Lo Presti D, Pisani I, Gagliardi G, Scala RL, Novelli GP (2015). Assessment of total vascular resistance and total body water in normotensive women during the first trimester of pregnancy. A key for the prevention of preeclampsia. Pregnancy Hypertens.

[56] Tomsin K, Vriens A, Mesens T, Gyselaers W (2013). Non-invasive cardiovascular profiling using combined electrocardiogram-Doppler ultrasonography and impedance cardiography: An experimental approach. Clin Exp Pharmacol Physiol.

[57] Tomsin K, Mesens T, Molenberghs G, Gyselaers W (2012). Venous pulse transit time in normal pregnancy and preeclampsia. Reprod Sci.

[58] Easterling TR, Benedetti TJ, Schmucker BC, Carlson K, Millard SP (1991). Maternal hemodynamics and aortic diameter in normal and hypertensive pregnancies. Obstet Gynecol.

[59] Vinayagam D, Patey O, Thilaganathan B, Khalil A (2016). Non-invasive cardiac output monitoring in pregnancy: comparison to echocardiographic assessment. Ultrasound Obstet Gynecol.

[60] Roberge S, Villa P, Nicolaides K, Giguere Y, Vainio M, Bakthi A (2012). Early administration of low-dose aspirin for the prevention of preterm and term preeclampsia: a systematic review and meta-analysis. Fetal Diagn Ther.

[61] Bujold E, Roberge S, Lacasse Y, Bureau M, Audibert F, Marcoux S (2010). Prevention of preeclampsia and intrauterine growth restriction with aspirin started in early pregnancy: a meta-analysis. Obstet Gynecol.

[62] Harrison CL, Brown WJ, Hayman M, Moran LJ, Redman LM (2016). The Role of Physical Activity in Preconception, Pregnancy and Postpartum Health. Semin Reprod Med.

[63] Waugh J, Bosio P, Habiba M, Boyce T, Shennan A, Halligan A (2001). Home monitoring of blood pressure in pregnancy at high risk of pre-eclampsia. Eur J Obstet Gynecol Reprod Biol.

[64] Duley L, Henderson-Smart DJ, Meher S, King JF. Antiplatelet agents for preventing pre-eclampsia and its complications. Cochrane Database Syst Rev. 2007;CD004659. doi: 10.1002/14651858.CD004659.pub2. 10.1002/14651858.CD004659.pub2 17443552

[65] Hofmeyr GJ, Lawrie TA, Atallah AN, Duley L. Calcium supplementation during pregnancy for preventing hypertensive disorders and related problems. Cochrane Database Syst Rev. 2010;CD001059. doi: 10.1002/14651858.CD001059.pub3. 10.1002/14651858.CD001059.pub3 20687064

